# A comparison of SARS-CoV-2 rapid antigen testing with realtime RT-PCR among symptomatic and asymptomatic individuals

**DOI:** 10.1186/s12879-022-07969-0

**Published:** 2023-02-09

**Authors:** Jyotsnamayee Sabat, Subhra Subhadra, Sonalika Rath, Lal Mohan Ho, Tanushree Satpathy, Dipankar Pattnaik, Sanghamitra Pati, Jyotirmayee Turuk

**Affiliations:** 1 Virus Research and Diagnostic Laboratory (VRDL), ICMR-Regional Medical Research Centre, Chandrasekharpur, Bhubaneswar, Odisha 751023 India; 2 East Coast Railway Hospital, Bhubaneswar, Odisha India; 3 Department of Microbiology, VIMSAR, Burla, Sambalpur, Odisha India

**Keywords:** RAT, SARS-CoV-2, RT-PCR, Specificity, Sensitivity, Symptomatic

## Abstract

**Background:**

Identification of SARS-CoV-2 positive patients with rapid and cost-effective test methods is the key for isolating infected individuals, interrupting the transmission chain, and thus, containment of the CoVID-19 disease. In this regard, Rapid Antigen Test (RAT) plays an important role at point of care testing but the low sensitivity attributing towards escape of positive cases is reported as a major disadvantage of RAT which led us to evaluate a RAT kit among symptomatic and asymptomatic individuals suspected of CoVID-19.

**Methods:**

We analyzed 329 parallel nasopharyngeal swabs for RAT (Zydus Cadila, India) at the point of collection in a hospital-based facility and RealTime RT-PCR in the laboratory. The performance parameters were analyzed by evaluating the specificity, sensitivity, Negative Predictive Value (NPV), Positive Predictive Value (PPV), and Kappa coefficient.

**Results:**

The sensitivity and specificity were found to be 75.17% and 98.89% respectively. Positive Predictive value was 98.25% and the negative predictive value was 82.79%. The accuracy between the two techniques was found to be 88.14% with a kappa coefficient of 0.756 (SE: 0.036 and CI at 95%: 0.686 to 0.826) with a good strength of agreement (0.61–0.80) between the two testing techniques. Among the false-negative cases, 22 (59.5%) were asymptomatic having the Cycle Threshold (Ct) range 27 to 32.9 including 12 cases with a history of close contact with the known positive cases (i.e. household contact). The remaining 15 cases (40.5%) were symptomatic having low to moderate Ct values.

**Conclusion:**

It is observed from the results that the false negative result for symptomatic individuals is a matter of concern as it was noted in 4 cases of our study subjects who required hospitalisation later. Also the positives among asymptomatic contacts are important from epidemiological point of view for isolation and curtailing the infection from spreading in a community. These results support the fact that RAT showing sensitivity below 80% can be used for mass screening purposes with provision for additional testing in case of false negative with symptomatic individuals. Also false-negative results should be interpreted cautiously considering the epidemiological link as well as the clinical condition of the patients.

## Background

COVID-19 Pandemic has posed a public health challenge not only for India but also for the other countries in the world in terms of mass testing and containment. RealTime RT-PCR is considered as the gold standard test method for laboratory diagnosis of SARS-CoV-2 due to its high specificity and sensitivity. Most of the laboratories that rely on RealTime RT-PCR for detection of SARS-CoV-2 face limitations like the requirement of costly chemicals, logistics for transportation of samples, and result turn-around-time (TAT) [1]. This has posed a lot of challenges for mass and immediate screening followed by containment of infected individuals. RAT at point of care is useful where hospital and testing laboratories are not accessible posing a challenge for public health measures [2, 3]. In most of the cases it is inexpensive when compared to RealTime RT-PCR without requiring much expensive or specific instruments. Mass testing can be done within a short period of time that will help to take immediate public health measures and interrupt the chain of infection. Data from a meta-analysis has shown that the average specificity and sensitivity of RAT for SARS-CoV-2 were 99.5% and 56.2% respectively [4]. As per WHO, RAT is the first choice for mass screening in most of the countries in the world because of easy accessibility, immediate result, contact tracing and implication of interventional measures [5]. Most of the evaluations for RAT-based testing have been done on stored samples which may not give accurate results when compared to clinical conditions [6]. Some studies have been done on symptomatic and asymptomatic individuals indicating its mass testing and epidemiological implication [3].

A cost effective RAT kits having good specificity and sensitivity is an useful tool for large-scale screening purposes. Comparison between RAT performance with respect to RealTime assay is essential to prevent missing out of false-negative results. There are as many as 50 RAT kits in the list recommended by Indian Council of Medical Research (ICMR) in India as of July 2021 having a wide range of sensitivity and specificity [7].

The usefulness of RAT is a question mark where the disease prevalence is low especially for the kits having low sensitivity. Also limited studies are there to establish the fact that the asymptomatic individuals tested negative by RAT but positive through RealTime PCR could spread the infection. Most of the studies have been conducted on either symptomatic or asymptomatic individuals. This study was focused on comparison between RAT and RealTime RT-PCR assay among both symptomatic and asymptomatic individuals with an emphasis on disease condition and hospitalization. Based on these comparisons suggestions can be made whether RAT alone, despite its low sensitivity can be used for mass screening at the point of care testing, and if so what would be the effect on clinical intervention.

## Methods

### Study area and clinical samples

This study was performed at Regional Virus Research and Diagnostic Laboratory, Indian Council of Medical Research (ICMR), Regional Medical Research Centre, Bhubaneswar, Odisha, India in 2021. This laboratory is the apex referral laboratory for testing and quality control of COVID-19 samples in the state of Odisha. We coordinated for collection of a total of 329 nasopharyngeal swabs (in duplicates) from five different tertiary care hospital setups/medical colleges located at the central and western part of Odisha. A detailed history of clinical signs and symptoms were collected in pre-designed proforma during sample collection.

Symptomatic cases were divided into two categories, one who required hospitalization for treatment and the other category included those who experienced mild symptoms and stayed in home care with general medication.

Nasopharyngeal samples using nylon swabs were collected in the Viral Transport Media (VTM, Make-HiMedia, India). One swab sample was immediately used for testing of SARS-CoV-2 by using a RAT kit (Zydus Cadila, India) at the hospital/medical college where sample was collected. This RAT kit is based on the principle of rapid lateral flow immunoassay and is designed to give results within 30 min for SARS-CoV-2 nucleoprotein. The kit contains all required reagents and a testing manual. The kits are stable when stored at room temperature as provided in the manufacturer’s instruction manual. The second swab sample in VTM was transported to the laboratory maintaining 4 °C and stored at − 80 °C till RNA extraction was done.

### RNA extraction and RT-PCR

RNA extraction was done by using a magnetic bead extraction kit from Mag Max Viral/Pathogen nucleic acid isolation kit (Thermo Fisher Scientific, Waltham, MA, USA) on the Kingfisher Flex Magnetic automated extractor (Thermo Fisher Scientific). A total of 5 μL proteinase K, 265 μL binding solution, 10 μL total nucleic acid-binding beads were mixed thoroughly and added to each well of a deep-well 96-well plate containing 200 μL of VTM (Sample). Another 3 plates containing 500 μL wash buffer, 500 μL 80% ethanol and 50 μL elution buffer separately were kept along with the sample plate and allowed for extraction. Extracted RNA was obtained in the elution plate containing 50 μL of elution buffer. RT-PCR was performed on RealTime PCR. The RT-PCR kit used was the TaqPath™ COVID-19 RT PCR Kit and the procedure was followed as per the instruction manual provided along with kit (Cat No. A47817, Thermo Fisher Scientific, USA). The kit targets genes of Nucleo-capsid Protein (N), Surface Protein (S), and ORF1 ab (ORF).

RT-PCR was performed using the TaqPath™ COVID-19 RT PCR Kit (Cat No. A47817, Thermo Fisher Scientific, USA) as per the manufacturer’s instruction in ABI-7500 Fast RealTime instrument. The target genes N, S, and ORF-1 were multiplexed and acquired in VIC, ABY, and FAM channels respectively. JUN was used as internal control (MS2) and run in the same reaction. The signal thresholds for each target were set manually and the Ct value less than 35 were considered as detected. The sample was considered as positive if two or more targets were detected with a positive MS2 result. Sample was considered as negative if no target was detected with a positive MS2 result. In case of target detected in one gene with a positive/negative MS2 result, the sample was considered as inconclusive and a repeat testing was done. The sample was considered as invalid if no target was detected along with a negative result of MS2. The Ct value was the mean value of both N and S genes considered for result calculation in case of a positive sample. The Cycle threshold for each target was manually adjusted and the Ct value less than 35 were considered as positive.

The study was conducted as per standard guidelines of GCP (Good Clinical Practice) & GLP (Good Laboratory Practice). Approval of the human ethical committee was obtained. Written informed consent from the patients/guardians was taken before enrolment and sample collection. The report of the laboratory diagnosis was provided to the patients/treating physician for patient care in free of cost on ethical ground.

### Statistical analysis

Data were entered and analysed in the Microsoft Excel for percentage calculation. Statistical analysis considered sensitivity, specificity, Positive Predictive Value (PPV), and Negative Predictive Value (NPV), accuracy, Kappa coefficient, by taking Confidence Interval at 95% were calculated by using MedCalc software.

## Results

A total of 329 patients were enrolled with or without the symptoms. Most of the asymptomatic individuals were from close contacts of COVID-19 disease positive patients. Patients were belonged to mostly from eastern and western part of the state. While attending OPD, cases were presented with mild to moderate grade of symptoms.

Samples were tested for performance evaluation for both antigen and RealTime RT-PCR among symptomatic as well as asymptomatic individuals. Rapid antigen test was performed right after sampling of nasopharyngeal swab at the point of collection where as RealTime assay was performed in the main laboratory. The cases included 233 (70.8%) of males and 96 (29.2%) of females. The majority of patients belonged to 19–45 years of age range (71.4%) followed by 46–60 (20%) years of age group. Among the total cases, 141 (42.8%) were asymptomatic and 188 (57.2%) were symptomatic having one or more symptoms pertinent to COVID-19 disease. Most symptomatic cases were present in the 19–45 years of age range presenting 75% (n = 141) of the total symptomatic cases. The demographic details of the study population are presented in Table 1.

**Table 1:** Demographic details of the patients included in the study (n = 329)

Parameters	Symptomatic	%	Asymptomatic	%	Total	%
Sex						
Male	141	42.9	92	28.0	233 (Male)	70.8
Female	47	14.3	49	14.9	96 (Female)	29.2
Age group (Years)						
≤ 18	3	0.9	6	1.8	9 (≤ 18)	2.74
19–30	70	21.3	48	14.6	118 (19–30)	35.9
31–45	71	21.6	46	14.0	117 (31–45)	35.6
46–60	31	9.4	35	10.6	66 (46–60)	20.1
> 60	13	4.0	6	1.8	19 (> 60)	5.78

Among all the samples tested, 114 (34.6%) were antigen-positive and 149 (45.2%) were RT-PCR positive. Out of all tested through both the methods, 112, 178,2 and 37 were true positive, true negative, false positive and false negatives respectively. The antigen testing sensitivity was found to be 75.17% (112 out of 149) and specificity was 98.89% (178 out of 180).The positive predictive value (PPV) was 98.25% and the negative predictive value (NPV) was 82.79%. The accuracy between the two techniques was found to be 88.14% with a kappa coefficient of 0.756 (SE: 0.036 and CI at 95%: 0.686 to 0.826) indicating a good strength of agreement (0.61–0.80) between the two techniques (Table 2).

**Table 2:** Comparison for sensitivity and specificity between RAT and real-time RT-PCR test results

Rapid antigen test (RAT)	Real-time RT-PCR for COVID-19		Total
	Positive	Negative	
Positive	112	2	114
Negative	37	178	215
Total	149	180	329

Performance parameters	Value	95% CI
Sensitivity	75.16%	67.43 to 81.87%
Specificity	98.89%	96.04 to 99.87%
PPV	98.25%	93.36 to 99.55%
NPV	82.79%	78.43 to 86.42%
Accuracy	88.15%	84.15 to 91.43%
Kappa	0.756	0.686 to 0.826
SE of kappa	0.036	

**Fig. 1:**
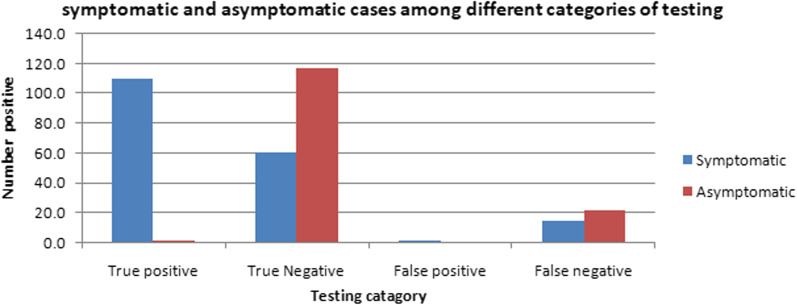
Distribution of symptomatic and asymptomatic cases among different categories of testing

The symptomatic cases included mild to severe disease category patients (n = 188) whereas hospitalization was required in 23 cases. Out of 112 true positive cases, 110 (98.2%) were symptomatic representing 58.5% (n = 188) of the total symptomatic cases and 2 were asymptomatic. Two cases were presented with mild disease and found to be false-positive. There were 178 cases of true negatives. Asymptomatic cases were found to be 65.7% (n = 117) among true negatives followed by false negatives (59.5%). Considerable symptomatic cases were also present in true negatives (34.3%) and false-negative patients (40.5%) (Fig. 1).

Assuming that viral load is negatively associated with Ct value, a comparison between true positive and false negative patients was done. Among the true positive cases, more cases were presented with symptoms like fever, cold, cough, body ache, loss of taste and smell, weakness etc. (n = 91) followed by patients who required hospitalization (n = 19). There was not much difference between both the category of patients as far as Ct value was concerned (lowest Ct-15.3 and highest Ct-31.7). Among 19 hospitalized cases, 10 cases were having one or more co-morbid conditions. Two asymptomatic and true positive cases having a Ct ranging from 27.4 to 30.6 were also found to be antigen positive. Among the false-negative cases, 22(59.5%) were asymptomatic having the Ct range 27 to 32.9 including 12 cases that had a history of close contact with confirmed positive case (household contacts). The remaining 40.5% were symptomatic having low to moderate Ct values (Table 3). Among the symptomatic cases, 4 patients were severely ill requiring hospitalization and the Ct values ranged from 25.3 to 30.6.

**Table 3:** Comparison between Ct value and disease condition of true positive and false negative cases

	Mild	Ct Range	Moderate	Ct Range	Hospitalised	Ct Range	Asymptomatic	Ct Range
True positive (n = 112)	8	17.3–29.7	83	15.3–31.7	19	17–30.7	2	27.4–30.6
False negative (n = 37)	8	21–27.7	3	26.5–32	4	25.3–30.6	22	27–32.9

## Discussion

In this study the assay performance of an antigen kit was compared with RealTime RT-PCR. But as the RT-PCR test is not a rapid test also it requires specialized laboratory and trained technicians so there is a need for rapid test kit that can be used for screening for asymptomatic individuals as well as symptomatic patients. The antigen kit used for this study has a specificity of 98.9% and sensitivity of 75.16% respectively. It can be useful at point of care testing with additional strategy of testing to minimize the risk of detecting more false negative report especially in the setting where RT-PCR test is not available. Due to low sensitivity it cannot replace RealTime RT-PCR for diagnosis and surveillance for SARS-CoV-2 [8]. Studies have reported an average sensitivity of 72.0% (95% CI 63.7–79.0%) in 37 evaluations and 58.1% (95% CI 40.2–74.1%) in 12 evaluations among symptomatic individuals and asymptomatic individuals respectively [4]. The PPV and NPV are quite promising while considering the kit for screening among symptomatic and asymptomatic individuals.

Studies have established that positive SARS-CoV-2 RT-PCR do not allow definitive conclusions whether the patient is still contagious or not. This can be partially attained by analyzing the cycle threshold (Ct) value from a RealTime RT-PCR assay depicting the particular amount of viral RNA in the sample and a comprehensive conclusion on the viral load and infectivity. There are some studies where the antigen was detected up to 33 Ct value considering it as cut off for infectivity and had good clinical correlation [9] in comparison to our kit which has detected up to 32 Ct as the higher cut-off value. Moreover, the test may be considered of specific benefit in subjects with short duration of onset of symptoms as viral load is more during the first days of infection. It was observed that symptomatic individuals (n = 22) with false negative RAT result having Ct values ≥ 27 may be less infectious [10, 11]. Also, there should be a testing algorithm to perform RealTime RT-PCR test in subjects with symptoms compatible with COVID-19 but a negative antigen result. Home isolation should be followed till the RT-PCR result is obtained. Further more, symptomatic individuals with a false negative result are a matter of concern as 4 of them got hospitalized due to severe illness and 11 were having mild to moderate disease conditions in our study. Though they represented 4.5% (n = 15) of the total screened population, which is very low in proportion but cannot be ignored. Patients (n = 2) had flu like symptoms but negative by RT-PCR can be taken as possibly non specific.

We used RT-PCR results which is considered gold-standard to compare the RAT results instead of relying on clinical symptoms alone is the major strength of our study and the other strength is access to the patient hospitalization data, without which it would be not known the effect of the false positive/false negative results. The limitation of our study is that we have considered Ct-values instead of viral load quantification to define level of infectivity. Ct-values can vary considerably, either because of inconsistent sampling methods or the limit of detection of RT-PCR kits.

## Conclusions

RAT has a significant role in screening, testing, and contact tracing strategies that contribute to the control of the outbreak. As RAT is the first choice for mass screening at different places hence the sensitivity of the kit can be improved so that no positive case is declared negative and dependency on RealTime RT-PCR will be minimized. As per WHO recommendation the sensitivity of RAT kit needs to be > 80% to be used at point of care testing. The kit studied here has shown less sensitivity. It can be used with certain precautions like patients having onset of illness within 5 days which is the time when human-to-human transmission is more. Also the the patients having COVID like symptom but showing RAT negative should be retested through RealTime RT-PCR for further confirmation. Combination assay will help to estimate the infection phase of COVID-19 patients in routine clinical practice or surveillance. Also This study suggests that there is no specific relation between Ct value and antigen positivity/negativity. The clinical usefulness of using this kit among patients having > 32 Ct value is doubtful as there is higher probability of getting false negative results.

The clinical implication of RAT is important where RT-PCR is unavailable, the RAT can help in majority of the infection cases (75.17%) in comparison to 11.24% of cases were missed clinical care due to false negative result. In case of non severe cases a less accurate rapid result is often very useful and for more severe cases, a test might not be necessary, and should be treated based on the situation without waiting for a better or accurate laboratory result.

## Data Availability

The datasets generated and analysed during the current study are not publicly available due to limitations of ethical approval involving the patient data and anonymity but are available from the corresponding author on reasonable request.
